# Beyond Pain: Psychotherapeutic and Relational Factors in the Remission of Fibromyalgia Symptoms. Two Case Reports

**DOI:** 10.1192/j.eurpsy.2026.11737

**Published:** 2026-07-31

**Authors:** S. Castelao-Almodovar

**Affiliations:** Centro de Salud Mental El Escorial, El Escorial, Spain

## Abstract

**Introduction:**

Fibromyalgia and chronic fatigue syndrome are commonly associated with persistent somatic symptoms, significant functional impairment and limited response to pharmacological treatment. Complete remission of fibromyalgia-related pain is rarely described in clinical practice. Recent evidence highlights the interplay between psychosocial stressors, relational dynamics and somatic symptomatology, but reports of patients achieving a sense of “cure” remain exceptional.

**Objectives:**

To describe the clinical course of two female patients with fibromyalgia who experienced a remarkable remission of pain and fatigue symptoms, in temporal association with the exploration of relational conflicts and biographical narratives during psychiatric follow-up.

**Methods:**

We present two case reports (aged 62 and 64) followed in outpatient psychiatric consultations. Both had long-standing fibromyalgia and depressive-anxious symptoms treated with duloxetine and adjunctive agents. During the last year, beyond pharmacological adjustments, psychotherapeutic interventions focused on linking relational patterns and life history to bodily symptoms, encouraging expression of emotions and establishing interpersonal boundaries.

**Results:**

Both patients reported progressive disappearance of diffuse fibromyalgia pain and fatigue, contrasting with the persistence of mechanical pain due to musculoskeletal disorders (arthrosis, spine pathology). They spontaneously differentiated this residual pain from their previous fibromyalgia pain (“plof”/diffuse body pain). They associated the improvement with being able to verbalize anger and frustration, set limits in family or marital contexts, and recognize the impact of unresolved relational conflicts on somatic expression. Although pharmacological regimens included antidepressants previously used with partial effect, patients emphasized the transformative role of emotional expression and relational change. At present, both report full functional recovery and describe themselves as “cured” of fibromyalgia.

**Conclusions:**

These cases suggest that, in certain patients, remission of fibromyalgia symptoms may be facilitated by psychotherapeutic work addressing relational dynamics and biographical conflicts. Beyond pharmacological treatment, the ability to connect emotions with somatic experience, express conflict and set interpersonal boundaries may play a central role in the disappearance of chronic pain. Further research is needed to explore the mechanisms underlying these exceptional clinical evolutions.

**Disclosure of Interest:**

None Declared

